# NatHER: protocol for systematic evaluation of trends in survival among patients with HER2-positive advanced breast cancer

**DOI:** 10.1186/s13643-015-0118-z

**Published:** 2015-10-01

**Authors:** Eli J. Korner, Anne Morris, Isabel Elaine Allen, Sara Hurvitz, Mary S. Beattie, Bindu Kalesan

**Affiliations:** Genentech, Inc., 1 DNA Way, South San Francisco, CA 94080 USA; PRO Unlimited, Inc., 1350 Old Bayshore Hwy Suite 350, Burlingame, CA 94010 USA; College of Social and Behavioral Sciences, School of Psychology, Walden University, 155 5th Avenue South, Minneapolis, MN 55401 USA; Department of Epidemiology and Biostatistics, University of California, San Francisco School of Medicine, 550 16th Street, 2nd Floor, San Francisco, CA 94158 USA; UCLA Jonsson Comprehensive Cancer Center, 2020 Santa Monica Blvd., Suite 580, Santa Monica, CA 90404 USA; Department of Epidemiology, Columbia University, 722 W 168th Street, New York, NY 10032 USA; Pharmaceutical Product Development, 980 Harvest Drive #130, Blue Bell, PA 19422 USA

**Keywords:** Advanced breast cancer, HER2-positive breast cancer, Interventional controlled trials, Locally advanced breast cancer, Meta-analysis, Metastatic breast cancer, Observational study, Randomized clinical trial, Survival, Systematic review

## Abstract

**Background:**

Human epidermal growth factor receptor 2 (HER2)-positive metastatic breast cancer (MBC) is an aggressive form of breast cancer and is historically associated with poor outcomes compared with HER2-negative MBC. Since 1998, four drugs have been globally approved for the targeted treatment of HER2-positive MBC. Additional advances in patient care—such as improved breast cancer screening, HER2 testing, and supportive care—have also occurred. The objective of this systematic review and meta-analysis is to determine whether there has been a cumulative change in survival over time in patients with HER2-positive advanced breast cancer based on results from interventional clinical trials (ICTs) and observational studies and to compare outcomes across these types of studies.

**Methods/Design:**

A systematic search of Medline, EMBASE, and the Cochrane Central Register of Controlled Trials will be performed. Two investigators will independently assess each abstract for inclusion. English language reports of ICTs and observational studies that include patients with HER2-positive advanced breast cancer from 1987 onwards will be considered. The primary outcome of interest is overall survival; secondary outcomes include progression-free survival and safety. Data on clinical outcomes, as well as on study design, study population, treatment/intervention, methodological quality, and outcomes, will be extracted using a structured codebook developed by the authors for this study. Standard and cumulative random effects meta-analysis will be performed to derive pooled risk estimates, both overall and by study design, controlling for covariates such as aggregate demographic and clinical characteristics of patients, treatment/intervention, and study characteristics. Heterogeneity of studies will be evaluated using the I^2^ statistic. Differences in risk estimates by quality characteristics will be performed using meta-regression.

**Discussion:**

This study will evaluate current and evolving trends in survival associated with HER2-positive advanced breast cancer over nearly 30 years and will build upon prior, less comprehensive, systematic analyses. This information is important to patients, healthcare providers, and researchers, particularly in the advanced disease setting, in which new therapies have been recently approved. Including observational studies allows us to evaluate real-world effectiveness; useful information will be gained by comparing findings from observational studies with those from ICTs.

**Systematic review registration:**

PROSPERO CRD42014014345

**Electronic supplementary material:**

The online version of this article (doi:10.1186/s13643-015-0118-z) contains supplementary material, which is available to authorized users.

## Background

Breast cancer is the most common cancer among women worldwide. Globally, there were an estimated 1.67 million new breast cancer diagnoses and 522,000 breast cancer-related deaths in 2012 [1]. Human epidermal growth factor receptor 2 (HER2) is overexpressed in 15–20 % of all primary breast tumors [2–4]. Overexpression of HER2 is associated with indicators of more aggressive disease, such as positive lymph nodes and high nuclear grade [5–8]. Consistent with this, prior to the availability of HER2-targeted therapy, patients with HER2-positive breast cancer experienced significantly shorter disease-free survival [5, 6, 9] and an approximately twofold increase in breast cancer mortality [10–12] relative to patients with HER2-normal breast cancer.

The first HER2-targeted therapy, the humanized monoclonal antibody trastuzumab, was approved for the treatment of patients with metastatic breast cancer (MBC) in 1998. Since then, three additional HER2-targeted agents have been approved: the tyrosine kinase inhibitor lapatinib, the humanized monoclonal antibody pertuzumab, and the antibody–drug conjugate trastuzumab emtansine (T-DM1). In addition to these therapies, other advances in the care of patients identified with HER2-positive advanced breast cancer (i.e., MBC or locally advanced breast cancer [LABC]) have occurred, such as improvements in breast cancer screening, advances in reliable identification of HER2-positive disease, refinement of interventional approaches, and improvements in supportive care.

In the most comprehensive systematic review of HER2-targeted therapy to date, Giordano and colleagues evaluated all comparative phase III randomized trials, systematic reviews, and meta-analyses of patients with HER2-positive advanced breast cancer published through October 2012 [13]. The analysis found that HER2-targeted regimens were associated with improvements in both progression-free survival (PFS) and overall survival (OS) relative to chemotherapy alone. This analysis, however, did not evaluate potential changes in the magnitude of the improvements in PFS and OS over time but focused, instead, on the collective impact of HER2-targeted therapies. While a different systematic review did set out to define changes observed in randomized clinical trials in survival over time, including in studies of patients with HER2-positive advanced breast cancer, this systematic review included only studies that assessed trastuzumab-based therapy [14]. With multiple HER2-targeted treatments now available, there is a need to analyze all available data in a comprehensive way.

While no comprehensive systematic reviews of potential changes in outcomes with HER2-targeted therapy over time in randomized clinical trials are currently available, data from historical versus current phase III randomized clinical trials suggest that survival outcomes may be changing. For example, from June 1995 to March 1997, the phase III trial that supported the licensure of trastuzumab recruited patients with HER2-positive MBC (including patients with both HER2 immunohistochemistry (IHC) 3+ and IHC 2+ tumors) who were not previously treated in the metastatic setting. Median OS among patients who received trastuzumab plus chemotherapy was 25.1 months compared with 20.3 months in the control arm [15]. Recruitment for the phase III CLEOPATRA study occurred between February 2008 and July 2010 [16]. The study included patients with HER2-positive advanced breast cancer (IHC 3+ or amplification ratio ≥2.0 by fluorescence in situ hybridization) not previously treated in the metastatic setting. Median OS was 40.8 months in the control arm (trastuzumab plus docetaxel) and 56.5 months in the pertuzumab arm (pertuzumab plus trastuzumab plus docetaxel) [17]. This comparison across clinical trials, however, is limited because of differences in study designs and patient populations. A comprehensive review and assessment of published interventional clinical trials is necessary to determine if a reliable change in outcomes exists.

Further, data from clinical trials do not always reflect findings in real-world clinical populations. To reduce potential biases in randomized clinical trials and other interventional trials, inclusion and exclusion criteria are applied. This can result in patient populations that are not universally generalizable to the routine clinical practice setting in which patients may have substantial differences in demographic characteristics, more comorbidities and concomitant medications, as well as complex psychosocial circumstances. In addition, patients enrolled in clinical trials tend to be higher functioning [18], and treatments/interventions and assessments are more uniform and more closely monitored. Observational studies may more closely approximate clinical practice in that these studies tend to have fewer inclusion criteria and less stringent assessment schedules. However, since treatments and assessments are generally less uniformly applied, data on treatment/intervention effects may be more variable. Further, observational studies may be more subject to certain types of biases, such as selection bias due to the absence of randomization [19].

There are limited published data on the collective effect of interventions to manage HER2-positive advanced breast cancer on outcomes in patients in real-world settings, and none of these systematically assess changes in survival outcomes over time. New data from a large epidemiological database of the California population, the California Cancer Registry, suggest that current survival outcomes for patients with HER2-positive MBC are similar to current survival outcomes for patients with HER2-negative MBC. This study, which included 6268 patients with MBC and 118,817 patients with early-stage breast cancer, found 3-year survival rates of 47.6 % for patients with HER2-positive MBC and 44.8 % for patients with HER2-negative MBC [20]. These similar 3-year survival rates stand in contrast with the negative prognosis associated with HER2-positive breast cancer that existed prior to the availability of HER2-directed therapy [5, 6, 9–12]. However, it cannot be concluded that the similar 3-year survival rates are due to HER2-directed therapy since observational studies can only show associations, rather than direct causes and effects. While these data suggest changes in real-world survival outcomes over time, it is necessary to comprehensively review and assess published observational studies to examine whether such a change has occurred.

Thus, limited evidence from both observational and randomized clinical trial data—as well as clinical experience—suggests that patients with HER2-positive advanced breast cancer have experienced improved survival outcomes over time. However, to our knowledge, no prior research has examined this by systematically assessing available phase II and III interventional clinical trial and observational study data, regardless of treatment/intervention, over a 30-year period. It is particularly important to assess the clinical implications of data from interventional clinical trials by investigating similar outcomes in the real-world setting, in which patients may have concomitant disease and more negative prognostic factors than those in interventional clinical trial populations. The use of real-world data and observational study designs involving data obtained from secondary data sources, including large population-level data sets, is increasing. Analyzing how patient characteristics and outcomes are similar or different to interventional controlled trials is pivotal to better understand and interpret both the data obtained from interventional controlled trials and those from observational data sources.

To examine survival outcomes in patients with HER2-positive advanced breast cancer in a comprehensive way, we will conduct a systematic literature review and meta-analysis. Our study will systematically identify and review available published data from phase II and III interventional clinical trials and observational studies that reported survival outcomes in patients with HER2-positive advanced breast cancer from 1987, when HER2-overexpressing breast cancer was identified as a distinct phenotype, to the present. Here, we describe the protocol for this systematic review and meta-analysis.

### Objectives

We will systematically evaluate clinical outcomes over time in patients with HER2-positive advanced breast cancer from both interventional clinical trials and observational studies and compare patient characteristics, study characteristics, and outcomes within and between these study design types.

## Methods/Design

### Study registration

This protocol has been registered with the international prospective register of systematic reviews (PROSPERO) as number CRD42014014345 (http://www.crd.york.ac.uk/PROSPERO/display_record.asp?ID=CRD42014014345). The systematic review protocol has been designed, conducted, and reported by using the Preferred Reporting Items for Systematic Reviews and Meta-Analyses (PRISMA) guidelines, as detailed in Additional file 1, and the Meta-analysis of Observational Studies in Epidemiology (MOOSE) reporting guidelines [21, 22].

### Eligibility criteria

Eligible studies will meet the following criteria:

- Population: Adults with HER2-positive advanced breast cancer (i.e., HER2-positive MBC or LABC) identified prior to receipt of the first targeted or chemotherapeutic agent for advanced breast cancer. HER2-positive status is defined by each publication. Mixed populations in which some patients had HER2-positive MBC and others had LABC were permitted. When tumor, node, metastasis (TNM) status was provided, it was used to further evaluate the study population for inclusion. The TNM classifications of each subgroup are shown in Additional file 2. Only those studies with at least 15 patients with HER2-positive advanced breast cancer will be used for the final analysis.
- Intervention/comparator: Any (e.g., surgery, chemotherapy, hormonal therapy, radiation therapy, or no intervention).
- Outcomes: OS and PFS.
  o For both interventional controlled trials and observational studies, eligible studies defined OS as the time from the initiation of first-line treatment in the advanced setting or as the time from diagnosis of advanced breast cancer until death from any cause.
  o Eligible interventional controlled trials defined PFS as the time from randomization until objective tumor progression or death [23]. Patient deaths are assumed to be randomly related to tumor progression for the purpose of calculating PFS. Eligible observational studies define PFS as the time from the initiation of first-line treatment or diagnosis of advanced breast cancer until objective tumor progression or death.
- Time of follow-up: At least 1 year.
- Study design: Randomized or quasi-randomized controlled trials (i.e., interventional controlled trials) and observational studies identified in the published literature from 1 January 1987 to 5 September 2014. Interventional controlled trials will be limited to phase II/III clinical trials.

### Outcomes of interest

Primary outcome:

- The primary outcome of interest is OS in patients with HER2-positive advanced breast cancer, which will be evaluated both overall and separately for interventional controlled trials and for observational studies.

Secondary outcomes:

- PFS.
- Safety: The proportion of patients experiencing adverse events and serious adverse events, the types of adverse events reported, and the proportion of patients experiencing adverse events of particular interest as reported in prior studies (e.g., cardiac toxicity [24]).
- Response rates (when available).
- Comparative assessment of study characteristics.

### Search strategy for the identification of studies and methods of review

We will conduct a systematic search of Medline, EMBASE, and Cochrane Central Register of Controlled Trials to identify relevant studies published between 1 January 1987 and 5 September 2014. EMBASE will be used to identify conference abstracts. Abstracts with a publication date within 3 years from the data cutoff (i.e., 5 September 2014) are eligible for inclusion. The search will be updated 1 month prior to the first submission of any manuscript based on these data. The search terms in each of the bibliographic databases are detailed in Additional file 3. The search will be limited to humans and to English language abstracts. Duplicates will be removed, and two investigators will assess the abstracts independently by applying the inclusion/exclusion codes. These codes are outlined in Additional file 4. The kappa statistic will be used to calculate agreement between the two investigators [25, 26]. Abstracts that are rated discordantly will be reconciled through discussion by the two independent raters and, if needed, adjudicated by a third investigator. The full-text versions of all abstracts that are rated as potentially eligible by both investigators will be obtained to perform in-depth evaluations using inclusion/exclusion codes, as shown in Additional file 5. The final inclusion/exclusion status of each article will be summarized and presented in a PRISMA flow chart [21].

### Data extraction

Data extraction will be performed using a standardized data extraction tool developed for this study by the study team. Two independent reviewers with advanced clinical and methodologic expertise will extract data from each publication or congress abstract meeting inclusion criteria across the following domains: study characteristics, patient demographic and clinical characteristics, treatment/intervention, survival outcomes, safety events, and methodologic characteristics. Discrepancies in the coding of data will be resolved by the two independent reviewers in order to reach 100 % agreement, and if needed, a third study investigator will adjudicate.

### Study variables

We will extract the following data:

- Study characteristics: Author names, year of publication, study setting, sponsorship of study, type of study design, year of start of study enrollment or observation, year of enrollment end or end of observational period, maximum follow-up time, median follow-up time, and types of intervention reported (e.g., pharmacologic, surgical, radiation, none).
- Population characteristics: Treatment/intervention, number of participants enrolled in the study, age of study participants, race/ethnicity, Eastern Cooperative Oncology Group performance status, type of HER2 test and result (cutoff), HER2 assessment at local or central laboratory, number and proportion of patients with MBC and LABC, type of disease (de novo or recurrent), adjuvant treatment status, median disease-free interval, prior adjuvant therapies (including HER2-targeted therapy, chemotherapy, hormonal therapy, surgery, and radiologic therapy), hormone receptor status, sites of metastases (e.g., visceral, central nervous system, other), and proportion of patients who dropped out or who were lost to follow-up.
- Efficacy/effectiveness outcome variables: Number of deaths, median OS, median PFS, and 95 % confidence intervals, as well as event rates and numbers at risk at 1-, 2-, and 5-year intervals. Data from trial arms will not be excluded based on treatment/intervention type or lack of active treatment/intervention.
- Safety outcome variables: Proportion of patients experiencing adverse events and serious adverse events, types of adverse events reported, and proportion of patients experiencing adverse events of particular interest as reported in prior studies (e.g., cardiac toxicity [24]).

### Assessment of methodological quality

We will assess the methodological quality and potential bias in both interventional controlled trials and observational studies, using a structured measure of methodological quality developed and validated by Wao et al. [27]. This measure uses a checklist derived from relevant elements of existing evidence-based tools that include the Quality in Prognosis Studies tool [28], the Evidence-Based Medicine Group criteria for prognostic studies [29], the Newcastle-Ottawa Quality Assessment Scale [30], the Cochrane Collaboration risk of bias criteria [31], and other studies [32, 33]. The Wao et al. measure assesses potential bias in four domains: (1) participation bias (i.e., How well does the study sample represent the general population of interest?); (2) attrition bias (i.e., Is attrition balanced across outcomes or characteristics of interest?); (3) outcome measurement (i.e., Is the outcome of interest measured appropriately?); and (4) data analysis and reporting (i.e., Are the methods used optimal?). The checklist is comprised of 11 items for cohort studies and 14 items for randomized controlled trials. Each study is evaluated for each item and is assigned 1 point for each criterion that is adequately addressed. The overall methodological quality is determined by examining the proportion of relevant criteria fulfilled for studies within each design type (observational cohort, randomized controlled trial).

To ensure a thorough assessment of methodological quality in all included studies, we will employ further assessments, as described below.

Methodological quality of interventional controlled trials: We will assess concealment of allocation, determination of HER2 status at a central laboratory, independent assessment of PFS, and the inclusion of participants in the analysis according to the intent-to-treat principle. Concealment of allocation will be considered adequate if the investigators responsible for the selection of patients did not know prior to allocation which treatment was next in line (e.g., central randomization and sealed, opaque, sequentially numbered assignment envelopes). Any procedures based on predictable generation of allocation sequences and potentially transparent attempts to conceal allocation, such as assignment envelopes that were not opaque or not sealed, will be considered inadequate for randomized controlled trials [34]. The analysis will be considered to be according to the intent-to-treat principle if all patients were analyzed in the group to which they were originally assigned [35].

Methodological quality of observational studies: Due to inherent biases in observational designs, we will measure multiple methodological characteristics within each level. For cohort studies, we will include quality characteristics that will assess biases due to design, representativeness, comparability of groups, exposure, and outcome measure and attrition.

- Study design: We will note the presence or absence of comparison between at least two groups to assess the effect/association of an exposure and an outcome. We will also record whether exposure and outcome were registered concurrently (retrospective or prospective data collection; follow-up may be active or retrospective with no prospective assessment in a retrospective study).
- Representativeness: We will define this by the type of study inclusion (i.e., consecutive or random inclusion), criteria for justified exclusion of participants for analysis, and whether the results are generalizable (using Newcastle-Ottawa Scale) [36].
- Comparability: When there are two or more exposure groups, comparability will be determined by applying inclusion and/or exclusion criteria equally to all groups and inclusion of all enrolled patients in the analysis (analogous to the intent-to-treat principle used in randomized trials). Maintenance of comparability throughout the study will be assessed by whether the length of follow-up was similar between the groups [37].
- Biases in outcome measurement: We will assess this by using an explicit definition of outcome and by considering potential confounders in the study and objective assessment using uniform methods [38].
- Attrition: The effects of attrition will be assessed by reviewing dropout rates in the groups and, particularly, differential rates of attrition by participant characteristics [37, 39, 40].

For both interventional controlled trials and observational studies, we will also include study size, balance in the size of treatment/intervention groups, and primary funding source. Studies will be considered to have balanced sizes of treatment/intervention groups if the difference in the number of patients between groups was less than fourfold. Studies will be identified as “sufficient” if they included at least 15 patients in each treatment/intervention group and more than 50 patients overall. The funding information extracted will include setting (academic or not academic), industry sponsorship (fully industry-sponsored, industry-supported, or none), and any further details available on the role of the sponsor in the conduct of the trial (e.g., extent of industry involvement in the study, regardless of funding), as applicable.

### Data analysis

Aggregate data will be used to summarize time trends in OS and PFS for interventional controlled trials and observational studies. Individual study characteristics will be described and summarized separately.

The outcome data will be analyzed using random effects meta-analysis [41]. Random effects meta-regression models will be used to examine whether survival times are affected by type of study, time era, treatment/intervention (e.g., pharmacologic, surgical, radiation, none), size of study, funding source, demographic characteristics, clinical characteristics (e.g., hormone receptor status), world region, post-progression therapy, and relevant methodological quality parameters.

A cumulative meta-analysis will be performed to assess the potential shift in outcome values over time. A cumulative meta-analysis will be performed regardless of type of treatment/intervention (including none), followed by a subanalysis restricted by type of treatment/intervention [38]. We will use meta-regression to perform stratified analyses to establish the effect of the source of funding, whether or not analyses had been adjusted for relevant covariates, size of the study, and appropriate methodological characteristics.

For all meta-analyses, we will calculate the I^2^ statistic to calculate the amount of variation across studies that is attributable to heterogeneity rather than random variation [42]. We will construct funnel plots by plotting the natural logarithm of the relative risk of individual trials on the *x*-axis against their standard error on the *y*-axis. We will enhance funnel plots by including contours dividing the plot into areas of significance with a two sided *P* < 0.05 and areas of nonsignificance with a *P* ≥ 0.05 [43]. If studies appear to be missing in areas of nonsignificance, this will suggest the presence of bias. We will then assess funnel plot asymmetry with regression tests, a weighted linear regression of the natural logarithm of risk ratios on their standard errors [44]. All analyses will be performed using STATA 13.1 (Stata, College Station, TX, USA).

### Role of the study sponsor

This work represents a collaboration between the authors, who are a group of academic clinicians and researchers and representatives of the sponsor, Genentech. All authors participated in the development of the study design, drafting this article, and the decision to submit this protocol for publication. When the study described herein is conducted, data will be collected and analyzed by all authors, who will have primary responsibility for the completeness and accuracy of the data and analyses. While Genentech is sponsoring the study, the authors have primary responsibility for the data analysis and publication-related decisions. The authors intend to publish the results of their analysis and will make the final decision to submit the work for publication.

## Discussion

Characterizing the changes in survival outcomes over time in patients with HER2-positive advanced breast cancer will serve several purposes. First, this research may identify the extent to which changes in patient care have been associated with improvements in patient survival. Additionally, by including data from interventional controlled trials and observational studies, we hope to provide insight into how survival outcomes from these two different types of studies may differ. Results from interventional controlled trials provide an important assessment of the efficacy and safety of interventions. Observational studies provide additional insight into the effectiveness of treatments and other interventions in the setting of real-world patients, providers, and healthcare systems as they approximate this setting more closely than do interventional controlled trials. Current accurate assessments of OS can also inform clinical trial designs, as the result of a longer OS duration would necessitate trial designs with larger sample sizes and longer follow-up times. Finally, this study may contribute to the state of research knowledge about the characterization of HER2-positive advanced breast cancer over time by providing important historical and clinical context to current treatment approaches and outcomes for patients.

The inherent biases within different types of study designs present multiple challenges in meta-analyses of observational studies [45]. To help address the biases associated with observational studies, we will list potential biases so that the data can be interpreted in this context. We will also quantify the variation in outcomes across studies to identify potential outliers and determine if study design elements contributed to the aberrant data.

The selection of OS as the primary outcome of interest is a strength of our study. OS, when measured in intent-to-treat populations with large sample sizes, is described as the most reliable end point in oncology studies, least subject to bias in end point measurement, and often the preferred end point since blinding is not essential [23]. Assessment of OS is best performed using interventional controlled trials, minimizing biases by providing a direct outcome comparison group [23]. However, PFS is a tumor assessment outcome that does not require a large sample size, is censored at either tumor progression or death, and requires objective and quantitative assessment [23]. The assessment of both OS and PFS over time among patients with HER2-positive advanced breast cancer may also provide insight into potential differences observed in these two outcomes.

To our knowledge, this study will be the first comprehensive systematic review of outcomes in patients with HER2-positive advanced breast cancer that evaluates nearly 30 years of published data and includes both interventional clinical trials and observational studies, regardless of the presence or type of intervention. With the addition of more diagnostic and therapeutic interventions for patients with HER2-positive advanced breast cancer, it is important to patients, healthcare providers, and researchers to document and quantify current and evolving trends in outcomes. This is particularly important in the advanced setting, in which new therapies have been recently approved and to more fully understand the relationship between outcomes obtained in an interventional controlled trial setting relative to real-world clinical practice. It is thus essential that a rigorous protocol be designed to pool and systematically assess the available evidence.

## Additional files

Additional file 1:
**PRISMA-P Checklist.** This table provides a completed PRISMA-P checklist. (PDF 45 kb) Additional file 2:
**Tumor, lymph node, metastasis (TNM) classification for defining subgroups of patients with locally advanced breast cancer and metastatic breast cancer.** This table provides TNM classifications used in this study. (PDF 31 kb) Additional file 3:
**Search criteria for Medline, EMBASE, and CENTRAL.** This table shows search criteria for Medline, EMBASE, and CENTRAL. (PDF 69 kb) Additional file 4:
**Screening codes applied to abstracts.** This table shows screening codes applied to abstracts. (PDF 29 kb) Additional file 5:
**Screening codes applied to full texts.** This table shows screening codes applied to full texts. (PDF 30 kb)

## Abbreviations

HER2: human epidermal growth factor receptor 2
IHC: immunohistochemistry
LABC: locally advanced breast cancer
MBC: metastatic breast cancer
OS: overall survival
PFS: progression-free survival
MOOSE: Meta-analysis of Observational Studies in Epidemiology
PRISMA: Preferred Reporting Items for Systematic Reviews and Meta-analyses
TNM: tumor, lymph node, metastasis
