# Supplementary material for: NatHER: protocol for systematic evaluation of trends in survival among patients with HER2-positive advanced breast cancer
Source: Syst Rev. 2015 Oct 1;4:133. doi: 10.1186/s13643-015-0118-z (PMC4591587; doi:10.1186/s13643-015-0118-z)
Supplement: Additional file 1: — PRISMA-P Checklist. This table provides a completed PRISMA-P checklist. (PDF 45 kb) [file 13643_2015_118_MOESM1_ESM.pdf]

Table 1. PRISMA-P Checklist.

Topic	Item #	Description	Page Number
ADMINISTRATIVE INFORMATION			
Title			
Identification	1a	Identify the report as a protocol of a systematic review	Title and page 1
Update	1b	If the protocol is for an update of a previous systematic review, identify as such	Not an update
Registration	2	If registered, provide the name of the registry (e.g., PROSPERO) and registration number	Pages 1, 3
Authors			
Contact	3a	Provide name, institutional affiliation, and e-mail address of all protocol authors; provide physical mailing address of corresponding author	Pages 1, 7
Contributions	3b	Describe contributions of protocol authors and identify the guarantor of the review	Page 7
Amendments	4	If the protocol represents an amendment of a previously completed or published protocol, identify as such and list changes; otherwise, state plan for documenting important protocol amendments	This is not an amendment of a previously published protocol. Protocol amendments will be submitted to PROSPERO
Support			
Sources	5a	Indicate sources of financial or other support for the review	Page 7
Sponsor	5b	Provide name for the review funder and/or sponsor	Page 7
Role of sponsor/funder	5c	Describe roles of funder(s), sponsor(s), and/or institution(s), if any, in developing the protocol	Pages 6-7
INTRODUCTION			
Rationale	6	Describe the rationale for the review in the context of what is already known	Pages 2-3

Objectives	7	Provide an explicit statement of the question(s) the review will address with reference to participants, interventions, comparators, and outcomes (PICO)	Pages 1, 3-4
METHODS			
Eligibility criteria	8	Specify the study characteristics (e.g., PICO, study design, setting, time frame) and report characteristics (e.g., years considered, language, publication status) to be used as criteria for eligibility for the review	Pages 3-4
Information sources	9	Describe all intended information sources (e.g., electronic databases, contact with study authors, trial registers, or other grey literature sources) with planned dates of coverage	Page 4
Search strategy	10	Present draft of search strategy to be used for at least one electronic database, including planned limits, such that it could be repeated	Page 4<br>Additional files 2-5
Study records			
Data management	11a	Describe the mechanism(s) that will be used to manage records and data throughout the review	Page 4
Selection process	11b	State the process that will be used for selecting studies (e.g., two independent reviewers) through each phase of the review (i.e., screening, eligibility, and inclusion in meta-analysis)	Page 4
Data collection process	11c	Describe planned method of extracting data from reports (e.g., piloting forms, done independently, in duplicate), any processes for obtaining and confirming data from investigators	Pages 4-5
Data items	12	List and define all variables for which data will be sought (e.g., PICO items, funding sources), any pre-planned data assumptions and simplifications	Pages 4-5
Outcomes and prioritization	13	List and define all outcomes for which data will be sought, including prioritization of main and additional outcomes, with rationale	Pages 4, 6-7
Risk of bias in individual studies	14	Describe anticipated methods for assessing risk of bias of individual studies, including whether this will be done at the outcome or study level, or both; state how this information will be used in data synthesis	Pages 5-6

Data			
Synthesis	15a	Describe criteria under which study data will be quantitatively synthesized	Page 6
	15b	If data are appropriate for quantitative synthesis, describe planned summary measures, methods of handling data, and methods of combining data from studies, including any planned exploration of consistency (e.g., I2, Kendall's tau)	Page 6
	15c	Describe any proposed additional analyses (e.g., sensitivity or subgroup analyses, meta-regression)	Page 6
	15d	If quantitative synthesis is not appropriate, describe the type of summary planned	n/a
Meta-bias(es)	16	Specify any planned assessment of meta-bias(es) (e.g., publication bias across studies, selective reporting within studies)	Pages 5-6
Confidence in cumulative evidence	17	Describe how the strength of the body of evidence will be assessed (e.g., GRADE)	Pages 5-6
