# Supplementary material for: NatHER: protocol for systematic evaluation of trends in survival among patients with HER2-positive advanced breast cancer
Source: Syst Rev. 2015 Oct 1;4:133. doi: 10.1186/s13643-015-0118-z (PMC4591587; doi:10.1186/s13643-015-0118-z)
Supplement: Additional file 2: — Tumor, lymph node, metastasis (TNM) classification for defining subgroups of patients with locally advanced breast cancer and metastatic breast cancer. This table provides TNM classifications used in this study. (PDF 31 kb) [file 13643_2015_118_MOESM2_ESM.pdf]

Table 2. Subgroups of patients with locally advanced breast cancer (LABC) and metastatic breast cancer (MBC) will be defined as follows according to tumor, lymph node, metastasis (TNM) classification [1].

LABC	T	N	M
Stage IIb	T3	N0	M0
Stage IIIa	T0	N2	M0
	T1	N2	M0
	T2	N2	M0
	T3	N1	M0
	T3	N2	M0
Stage IIIb	T4	N0	M0
	T4	N1	M0
	T4	N2	M0
Stage IIIc	Any T	N3	M0
MBC	Any T	Any N	M1

Reference

1. American Joint Committee on Cancer. Breast Cancer Staging, 7th ed. https://cancerstaging.org/references-tools/quickreferences/Documents/BreastSmall.pdf. Accessed 25 Mar 2015.
