# Supplementary material for: NatHER: protocol for systematic evaluation of trends in survival among patients with HER2-positive advanced breast cancer
Source: Syst Rev. 2015 Oct 1;4:133. doi: 10.1186/s13643-015-0118-z (PMC4591587; doi:10.1186/s13643-015-0118-z)
Supplement: Additional file 3: — Search criteria for Medline, EMBASE, and CENTRAL. This table shows search criteria for Medline, EMBASE, and CENTRAL. (PDF 69 kb) [file 13643_2015_118_MOESM3_ESM.pdf]

Table 3. Search criteria for MEDLINE, EMBASE, and CENTRAL.

A. MEDLINE search criteria

**Step**	**MEDLINE**
**1**	(her2-positive or her2 positive).mp.
**2**	(HER2+ or HER2 positive).mp.
**3**	exp *Receptor, erbB-2/ or erb-2.mp.
**4**	epidermal growth factor receptor-2.m_titl.
**5**	epidermal growth factor receptor-2.mp.
**6**	exp *Receptor, Epidermal Growth Factor/ or epidermal growth factor receptor 2.mp.
**7**	1 or 2 or 3 or 4 or 5 or 6
**8**	exp Breast Neoplasms/
**9**	exp Breast/ or Breast Diseases/
**10**	Neoplasms/ or Adenocarcinoma/ or Carcinoma/
**11**	(brca or (breast adj4 (adenocarcinoma* or cancer* or carcinoma* or metasta* or neoplasm* or tumo?r))).mp. [mp=title, abstract, original title, name of substance word, subject heading word, keyword heading word, protocol supplementary concept word, rare disease supplementary concept word, unique identifier]
**12**	8 or 9 or 10 or 11
**13**	metasta*.mp
**14**	metastatic.mp. or exp *Neoplasm Metastasis/
**15**	secondary.mp.
**16**	spread.mp.
**17**	spreads.mp.

**18**	advanced.mp.
**19**	stage 4.mp.
**20**	stage IV.mp. or exp *Neoplasm Staging/
**21**	stage-IV.mp. or exp *Neoplasm Staging/
**22**	stage-4.mp.
**23**	13 or 14 or 15 or 16 or 17 or 18 or 19 or 20 or 21 or 22
**24**	12 AND 23
**25**	exp *Survival Analysis/ or exp *Survival/ or exp *Survival Rate/ or survival.mp.
**26**	progression-free survival.mp. or exp *Disease-Free Survival/
**27**	mortality.mp. or exp *Mortality/
**28**	death.mp. or exp *Death/
**29**	25 or 26 or 27 or 28
**30**	randomized controlled trial.pt.
**31**	controlled clinical trial.pt.
**32**	randomized controlled trial.sh.
**33**	random allocation.sh.
**34**	double blind method.sh.
**35**	single blind method.sh.
**36**	clinical trial.pt.
**37**	exp clinical trial/
**38**	(clin$ adj25 trial$).ti,ab.
**39**	((singl$ OR doubl$ OR trebl$ OR tripl$) adj25 (blind$ OR mask$)).ti,ab.
**40**	placebos.sh.

**41**	placebo$.ti,ab.
**42**	random$.ti,ab.
**43**	research design.sh.
**44**	comparative study.sh.
**45**	exp evaluation studies/
**46**	follow up studies.sh.
**47**	prospective studies.sh.
**48**	(control$ OR prospectiv$ OR volunteer$).ti,ab.
**49**	OR/30-48
**50**	Epidemiologic studies/
**51**	exp case control studies/
**52**	exp cohort studies/
**53**	Case control.tw.
**54**	(cohort adj (study or studies)).tw.
**55**	Cohort analy$.tw.
**56**	(Follow up adj (study or studies)).tw.
**57**	(observational adj (study or studies)).tw.
**58**	Longitudinal.tw.
**59**	Retrospective.tw.
**60**	Cross sectional.tw.
**61**	Cross-sectional studies/
**62**	OR/50-61
**63**	#49 or #62

**64**	7 AND 24
**65**	29 and 63 and 64
**66**	limit 65 to humans
**67**	limit 66 to English
**68**	limit 67 to yr="1986 -Current"

B. EMBASE search criteria (http://www.embase.com/home#/search:quickSearch/)

**Step**	**EMBASE**
**1**	her2 AND positive
**2**	'her2 positive'
**3**	'receptor erbb 2'
**4**	'erb 2'
**5**	'epidermal growth factor receptor 2'
**6**	'receptor epidermal growth factor'
**7**	#1 or #2 or #3 or #4 or #5 or #6
**8**	breast AND neoplasms
**9**	'breast neoplasms'
**10**	'breast diseases'
**11**	breast*
**12**	breast AND (adenocarcinoma* OR cancer* OR carcinoma* OR metasta* OR neoplasm* OR tumo?r)
**13**	breast AND (adenocarcinoma* OR cancer* OR breast AND tumor* OR breast AND tumour*)

**14**	#8 or #9 or #10 or #11 or #12 or #13
**15**	metasta*
**16**	metastatic
**17**	'neoplasm metastasis'
**18**	secondary
**19**	spread
**20**	spreads OR advanced
**21**	'stage 4' OR 'stage-4' OR 'stage iv' OR 'stage-iv' OR 'neoplasm staging'
**22**	#15 or #16 or #17 or #18 or #19 or #20 or #21
**23**	#14 AND #22
**24**	survival AND analysis
**25**	'survival analysis'
**26**	'survival/' OR 'survival rate/' OR 'survival'
**27**	'progression-free survival' OR 'progression free survival'
**28**	'disease-free survival' OR 'disease free survival'
**29**	'mortality' OR 'mortality/'
**30**	'death' OR 'death/'
**31**	#24 or #25 or #26 or #27 or #28 or #29 or #30
**32**	clinical AND trial
**33**	'clinical trial'/exp
**34**	'randomized controlled trial'/exp
**35**	'randomization'/exp

**36**	'single blind procedure'/exp
**37**	'double blind procedure'/exp
**38**	'crossover procedure'/exp
**39**	'placebo'/exp
**40**	randomized AND controlled AND trial$
**41**	rct.tw OR 'rct'
**42**	randomised AND controlled AND trial$
**43**	random AND allocation
**44**	randomly AND allocated
**45**	'allocated randomly'
**46**	'single blind$'
**47**	'double blind$'
**48**	'placebo$'
**49**	'prospective study'/exp
**50**	#32 or #33 or #34 or #35 or #36 or #37 or #38 or #39 or #40 or #41 or #42 or #43 or #44 or #45 or #46 or #47 or #48 or #49
**51**	'clinical study'/exp
**52**	'case control study'
**53**	'family study'/exp
**54**	'longitudinal study'/exp
**55**	'retrospective study'/exp
**56**	'prospective study'/exp
**57**	'randomized controlled trials'/exp
**58**	#56 NOT #57

**59**	'cohort analysis'/exp
**60**	'cohort study'
**61**	'cohort studies'
**62**	'cohort analysis'
**63**	'case control study' OR 'case control studies'
**64**	'observational study' OR 'observational studies'
**65**	'epidemiologic$ study' OR 'epidemiologic$ studies'
**66**	'cross sectional study' OR 'cross sectional studies'
**67**	#51 or #52 or #53 or #54 or #55 or #58 or #59 or #60 or #61 or #62 or #63 or #64 or #65 or #66
**68**	#50 or #67
**69**	#7 AND #23
**70**	#31 AND #68 AND #69
**71**	#70 AND [humans]/lim
**72**	#71 AND [english]/lim
**73**	#72 AND [1987-2014]/py

C. CENTRAL search criteria. (http://onlinelibrary.wiley.com/cochranelibrary/search/)

**Step**	**CENTRAL**
**1**	"Her2" or "HER-2" or "HER2-neu" or "her2/neu" or "Her2/neu" or "HER2/neu" or "Her-2-neu" or "HER-2-neu" or "her-2-neu" or "Her-2/neu" or "HER-2/neu" or "her-2/neu" or "Her-2neu" or "HER-2neu" or "her-2neu" or "Her2neu" or "HER2neu" or "her2neu"

**2**	"erb2 receptor" or "epidermal growth factor receptor" or "epidermal growth factor"
**3**	#1 or #2
**4**	MeSH descriptor: [Breast Neoplasms] explode all trees
**5**	'breast neoplasms'
**6**	'breast diseases'
**7**	breast*
**8**	breast and (adenocarcinoma* or cancer* or carcinoma* or metasta* or neoplasm* or tumo?r)
**9**	breast and (adenocarcinoma* or cancer* or breast and tumor* or breast and tumour*)
**10**	#4 or #5 or #6 or #7 or #8 or #9
**11**	MeSH descriptor: [Neoplasm Metastasis] explode all trees
**12**	"metasta*" or "metastatic" or "neoplasm metastasis" or "secondary" or "spread" or "spreads" or "advanced"
**13**	"stage 4" or "stage-4" or "stage iv" or "stage-iv" or "neoplasm staging"
**14**	#11 or #12 or #13
**15**	#10 and #14
**16**	MeSH descriptor: [Survival Analysis] explode all trees
**17**	"survival analysis" or "survival/" or "survival rate/" or "survival"
**18**	MeSH descriptor: [Disease-Free Survival] explode all trees
**19**	"disease-free survival" or "disease free survival"
**20**	"progression-free survival" or "progression free survival"
**21**	MeSH descriptor: [Mortality] explode all trees
**22**	MeSH descriptor: [Death] explode all trees

**23**	#16 or #17 or #18 or #19 or #20 or #21 or #22
**24**	#3 and #15
**25**	#23 and #24
**26**	Publication Year from 1987 to 2014
