# Supplementary material for: NatHER: protocol for systematic evaluation of trends in survival among patients with HER2-positive advanced breast cancer
Source: Syst Rev. 2015 Oct 1;4:133. doi: 10.1186/s13643-015-0118-z (PMC4591587; doi:10.1186/s13643-015-0118-z)
Supplement: Additional file 4: — Screening codes applied to abstracts. This table shows screening codes applied to abstracts. (PDF 29 kb) [file 13643_2015_118_MOESM4_ESM.pdf]

Table 4. Screening codes applied to abstracts.

Phase I codes (mutually exclusive):
c - Consider for inclusion during step 1, full text must be obtained
u - Unclear, full text must be obtained (choose if abstract is missing)
Exclusion codes, scored in order of appearance (hierarchical exclusion):
1 = Not HER2-positive advanced nor metastatic breast cancer
2 = Not interventional clinical trial and not observational study
3 = Neither OS nor PFS nor clinical response
4 = If interventional clinical trial or if observational study, but less than 1 year of follow-up (N/A for CR only)
5 = Editorial, comment, review, conference proceeding theme
6 = Systematic review or meta-analysis
7 = Double entry of references (same abstract, in different journals)
8 = Protocol of interventional clinical trial or observational study that fulfils inclusion
9 =Other, record
Other reason: ...

CR, complete response; HER2, human epidermal growth factor receptor 2; N/A, not applicable; OS, overall survival; PFS, progression-free survival.
