# Supplementary material for: NatHER: protocol for systematic evaluation of trends in survival among patients with HER2-positive advanced breast cancer
Source: Syst Rev. 2015 Oct 1;4:133. doi: 10.1186/s13643-015-0118-z (PMC4591587; doi:10.1186/s13643-015-0118-z)
Supplement: Additional file 5: — Screening codes applied to full texts. This table shows screening codes applied to full texts. (PDF 30 kb) [file 13643_2015_118_MOESM5_ESM.pdf]

Table 5. Screening codes applied to full texts.

Phase II codes using full text (mutually exclusive):
c - Consider for inclusion during step 1
a – Primary population is HER2-positive LABC and/or MBC
b – Primary population is HER2-positive breast cancer with extractable information for LABC/MBC subgroup
c – Primary population is LABC with extractable information for HER2-positive subgroup
u - Unclear, (choose ONLY if discussion is needed)
Exclusion use codes, scored in order of appearance (hierarchical exclusion):
1 = Population NOT as described in ca, cb or cc
2 = Not interventional clinical trial and not observational study (case series satisfying ca, cb, cc of sample size <25 to be indicated as exclusion 2)
3 = neither OS nor PFS nor clinical response
4 = If interventional clinical trial or if observational study, has <1 year of follow-up (N/A if clinical response is the only outcome)
5 = Editorial, comment, review, conference proceeding theme
6 = Systematic review or meta-analysis
7 = Double entry of references (same full text with or without minor differences, in different journals) (multiple reports on the same trial or observational cohort does not qualify as double entry)
8 = Protocol of interventional clinical trial or observational study that fulfils inclusion

HER2, human epidermal growth factor receptor 2; LABC, locally advanced breast cancer; MBC, metastatic breast cancer; N/A, not applicable; OS, overall survival; PFS, progression-free survival.
